# pH-Dependent Conformational Switch Impacts Stability of the PsbS Dimer

**DOI:** 10.1021/acs.jpclett.2c03760

**Published:** 2023-01-20

**Authors:** Maria Gabriella Chiariello, Fabian Grünewald, Rubi Zarmiento-Garcia, Siewert J. Marrink

**Affiliations:** ^ Groningen Biomolecular Sciences and Biotechnology Institute (GBB), University of Groningen, Nijenborgh 4, 9747 AG Groningen, The Netherlands

## Abstract

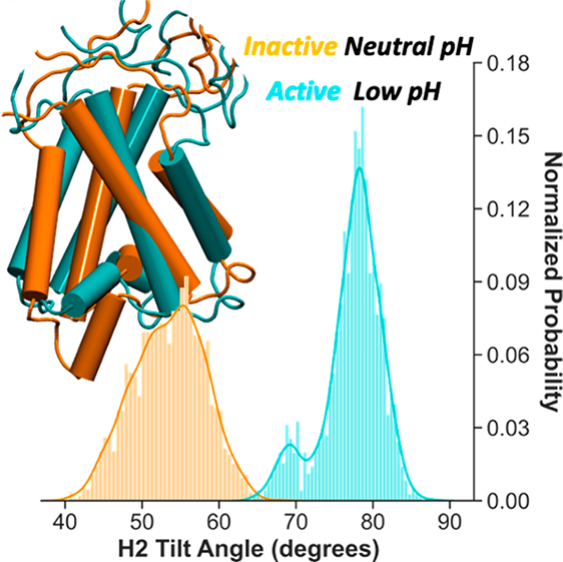

The photosystem II PsbS protein triggers the photoprotective mechanism of plants by sensing the acidification of the thylakoid lumen. Despite the mechanism of action of PsbS would require a pH-dependent monomerization of the dimeric form, a clear connection between the pH-induced structural changes and the dimer stability is missing. Here, by applying constant pH coarse-grained and all-atom molecular dynamics simulations, we investigate the pH-dependent structural response of the PsbS dimer. We find that the pH variation leads to structural changes in the lumen-exposed helices, located at the dimeric interface, providing an effective switch between PsbS *inactive* and *active* form. Moreover, the monomerization free energies reveal that in the neutral pH conformation, where the network of H-bond interactions at the dimeric interface is destroyed, the protein–protein interaction is weaker. Our results show how the pH-dependent conformations of PsbS affect their dimerization propensity, which is at the basis of the photoprotective mechanism.

In excess light, photosynthetic organisms absorb more solar energy than they require to perform useful photochemical reactions. This leads to the formation of lethal reactive oxygen species and severe damage to the cell. To prevent photodamage, the excess of harvested light energy is dissipated as heat through a phenomenon known as nonphotochemical quenching (NPQ).^1−3^

This photoprotective process is triggered by the low pH in the lumen of the photosynthetic thylakoid membrane.^4−6^ The excess of protons and the subsequent acidic environment are in turn created as subproducts of the water-splitting process catalyzed by the oxygen-evolving complex embedded in photosystem II (PSII).^7,8^

At the basis of the photoprotective process lies the response mechanism of the PsbS protein to the pH gradient established between the stromal (pH ∼ 7.5) and lumenal (pH ∼ 5.5) side of the membrane under excess light conditions.^4,9−11^ The PsbS is a small subunit of the light-harvesting (LHC) supercomplex,^12−14^ which acts as pH sensor.^15^ Cross-linking experiments show that the NPQ active/inactive states are characterized by different localizations of the PsbS within the LHC.^16^

In the current view, the PsbS dimeric form is inactive for NPQ,^17,18^ while the activation of the NPQ involves the monomerization of PsbS and enhanced interaction with the trimeric LHC.^5,16,19−21^ In this context, the switch between the monomeric and dimeric form of PsbS and the structural response of PsbS to the pH change are crucial and correlated aspects to understand the molecular basis of the NPQ. However, the link between monomer/dimer ratio and pH conditions is still elusive as conflicting evidence comes from different sources of experimental data. It was reported that the monomer is the prevalent form at low pH,^5^ but the dimer might exist at both low and neutral pH.^18^ Moreover, the only available X-ray structure for PsbS is a dimer obtained at pH 5;^22^ at neutral pH the dimer was found to be unstable in detergent solution.

Each PsbS monomer contains four transmembrane helices (TM1–4, see Figure 1A) and three short amphipathic helices (H1–3, highlighted in red in Figure 1A). Most of the structural response of the PsbS to the pH variation is localized to these amphipathic lumen-exposed helices.^23,24^ Each monomer contains 15 Glu residues, and eight of them (Glu 180, 182, 173, 69, 159, 55, 76, and 78) are exposed to the internal side. The X-ray structure (PDB code 4RI2) exhibits the key residue Glu 173 (which is part of H2) in hydrogen bond contact with Ile 74 and Tyr 75 (placed on H3) (see Figure 1A). Because H2 and H3 lie at the dimeric interface, the protonation state of Glu 173 can affect the stability of the dimeric structure. Moreover, mutations of Glu 173 and/or Glu 69 dramatically reduce the NPQ response.^15,25^ Recent FTIR and NMR experiments,^23^ as well as all-atom molecular dynamics (MD) simulations,^24^ suggest that the PsbS Glu residues are sensitive to the environmental conditions, undergoing a substantial change in protonation state when the pH drops from 7.5 to 5.0. The PsbS structural changes induced by the low pH are mainly (i) the variation of the secondary structure of H3 and (ii) the movement of H2 to the membrane phase.^23,24^ The pH-induced conformational change of the amphipathic helices H2 and H3 might reasonably have an impact on the dimeric stability. However, a clear connection between the pH-dependent conformations of PsbS and monomer/dimer equilibrium is still missing.

**Figure 1:**
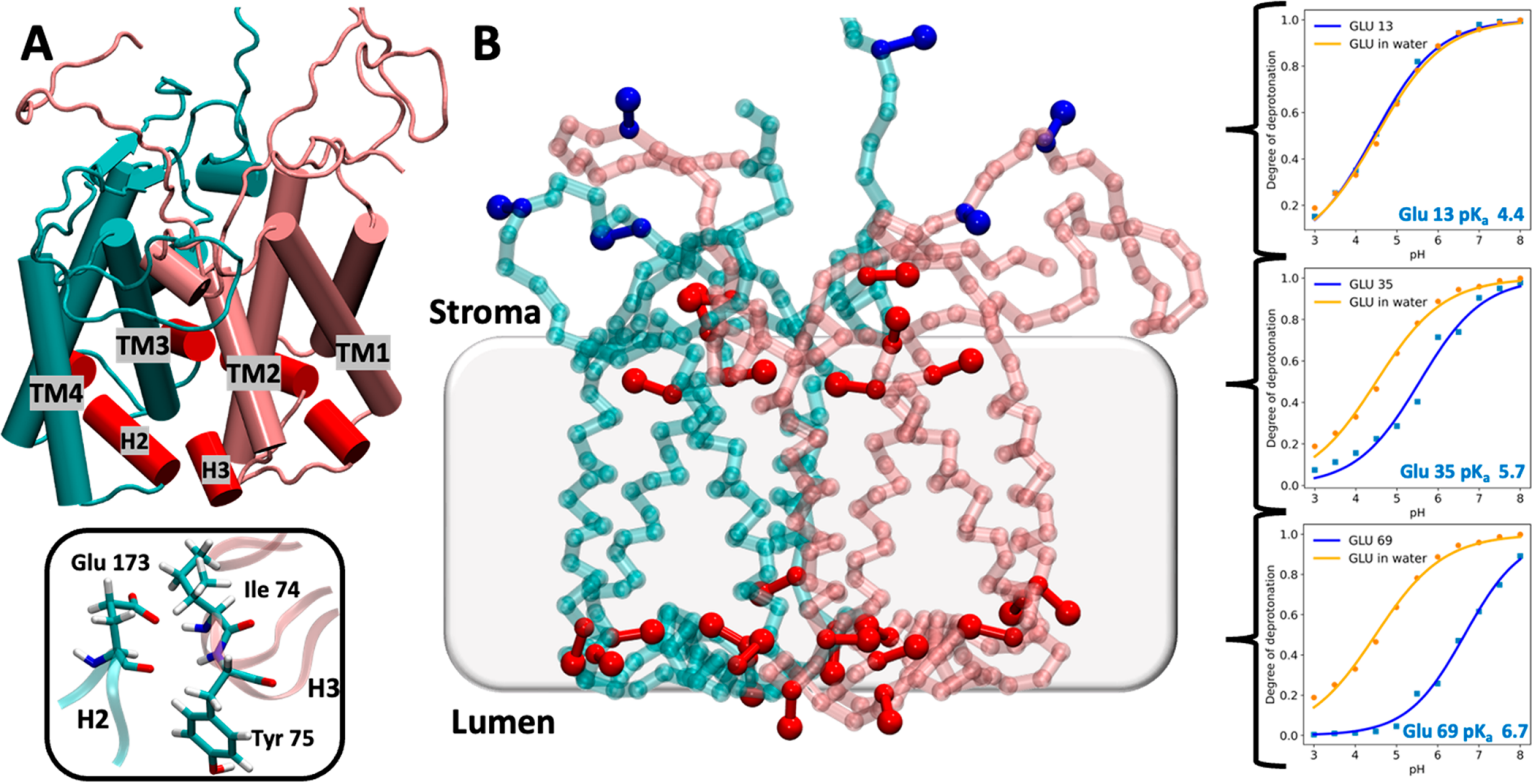
Shift of p*K*_a_ values of the Glu residues of PsbS dimer. (A) X-ray structure of the PsbS dimer a pH 5 (PDB code 4RI2). Each monomer contains four transmembrane helices (TM1–4) and three short helices (H1–3) facing the lumen. Helices H2 and H3 lie at dimeric interface and contribute to the dimer stability via H-bonds (inset). The structure, embedded in POPC and solvated with water and counterions, is used as starting configuration for the all-atom MD simulation. (B) Coarse-grained representation of PsbS dimer using Martini beads. The shady gray box approximately indicates the position of the POPC membrane. The Glu residues are explicitly shown in ball-and-stick representation. The Glu residues having a p*K*_a_ shifts between 0.6 and 3 respect to the standard p*K*_a_ value of Glu in water (4.5) are highlighted in red. A selected number of titration curves of Glu placed at the lumen (Glu69) and stromal (Glu35 and Glu13) are shown. The p*K*_a_ shifts can be appreciated by the overlap of the titration curves of Glu residues in PsbS (blue lines) and Glu in water (orange line) computed with the same method. Each point corresponds to the degree of deprotonation of the titratable site computed at a specific pH value. The curves are fitted using the Hill equation (see the Supporting Information).

Here, we use a multiscale computational workflow combining coarse-grained (CG) MD simulations based on the Martini force field,^26,27^ using a recently developed constant pH model,^28^ together with metadynamics (MTD)^29,30^ and additional simulations at the all-atom (AA) level to reproduce the structural response of PsbS to low pH and to quantify the effect of the different conformations and protonation states on the dimer dissociation energy. A schematic overview illustrating the workflow followed to perform all the simulations is given in Figure S1 and Table S1.

The initial CG model is based on the PsbS dimer X-ray structure embedded in 1-palmitoyl-2-oleoyl-*sn*-glycero-3-phosphocholine (POPC) bilayer. The additional elastic network potentials,^31−33^ underlying the CG model, are used to preserve the flexibility and the intrinsic dynamics of the protein with respect to the all-atom model (Figures S2 and S3). The equilibrated configuration is then used to perform the titration, i.e., the constant pH simulation. For this we use a recently proposed approach, Martini-sour, that allows to taking into account the effect of the pH within the Martini framework (see the SI Methods section for details about the protocol).^28^ Martini-sour can simulate a pH range of 3–8, which allows us to cover the pH gradient (from ∼7.5 to ∼5.5) relevant for the activation of NPQ. In our simulations, 15 Glu residues per PsbS monomer (Figure 1B) are considered titratable sites (Figure S4). We perform a total of 11 constant pH simulations, run for 1 μs each, covering the pH range 3–8. The degree of deprotonation is evaluated for all titratable sites at each pH value, and the p*K*_a_ value is obtained by fitting the resulting curve using the Hill equation (see SI eq 1). The p*K*_a_ shift induced by the protein environment is evaluated by comparison with the p*K*_a_ of glutamate in water,^34^ calculated with the same methodology (Figure S4).

The resulting titration curves of all the Glu residues of PsbS are depicted in Figures 1 and S5, and time-dependent switches in the protonation state are shown in Figure S6. Most residues experience a substantial p*K*_a_ shift, up to 3 p*K*_a_ units, compared to the Glu in water (p*K*_a_ = 4.5). Residues with a shift of >0.5 p*K*_a_ unit are highlighted in red in Figure 1B. The affected residues are found everywhere in the structure: exposed both to the lumenal (Glu 78, 76, 69, 55, 159, 173, 180, and 182) or stromal side (Glu 35, 20, and 37). The p*K*_a_ remains unshifted for three Glu placed on the stromal loop (Glu 13, 105, and 111) (Figures 1 and S5, Table S2). At pH 5 most of the Glu residues (12 out of 15) are either fully protonated or protonated for a significant amount of time (Figure S6), making the PsbS able to sense the pH range for the activation of NPQ. The p*K*_a_ shifts are consistent between the monomeric subunits and between different elastic network models (see Table S2 for the full list of computed p*K*_a_). Our findings are in nice agreement with recent solid-state NMR and FT-IR experiments,^23^ performed at different pH conditions, which suggest the protonation of most of the titratable acid residues of PsbS at pH 5. In the previous atomistic simulations of the PsbS monomer,^24^ only the residues exposed to the lumenal side (Glu 76, 78, 69, 141, 180, 182, and 173) were found to get protonated in the pH range 5–7^24^ (see Table S2). This discrepancy may be attributed to the limited time scale of the all-atom simulations, possibly preventing some residues from exploring different ways of embedding in the membrane in response to a change in protonation state due to an insufficient sampling of the rotational degrees of freedom of the amino acids side chains,^35^ and/or the difference in the oligomeric state, which could affect especially the affinity for protonation of the residues at monomer–monomer interface, such as Glu173.

Having established the p*K*_a_ shifts in the PsbS dimer, next we aimed to characterize possible structural changes of the protein in response to a change in protonation state. The CG resolution, however, does not allow to simulate specific conformational changes, such as folding or secondary structure variation. We, therefore, restore the atomistic resolution through a backmapping procedure^36^ of the CG structures obtained at pH 5.0 and 7.0 into a conventional (not titratable) atomistic model. To mimic the effect of the pH, the Glu residues are kept protonated according to the observed trend of p*K*_a_ shifts. In particular, the CG structure at pH 5 has been backmapped into an atomistic structure where 12 Glu residues including Glu173 (highlighted in red in Figure 1B) are protonated, while all of them are deprotonated in the atomistic model backmapped from pH 7. For each of these pH values (5 and 7) we extracted two configurations, and all the backmapped structures were simulated for 400 ns, after equilibration, using the CHARMM^37^ force field.

Analysis of the trajectories revealed an important difference between low and neutral pH conditions, namely a significant rearrangement of the H2 helix (Figure 2A,B). In Figure S7 we show the distribution and the time evolution of the tilt angle of H2 with respect to the membrane normal (*z*-axis) for both the monomers and the two replicas at neutral and low pH conditions, while the H2 tilt angle distribution obtained averaging over all the simulations is shown in Figure 2D. Here the angle explores values around 80° in the simulation at low pH, where the Glu residues are protonated. In these conditions, the H2 remains embedded in the membrane environment. In contrast, when all the Glu residues are deprotonated, i.e., at neutral pH conditions, the tilt angle distribution shows the peak at 50° corresponding to the movement of H2 into the solution. An overlap between the neutral and low pH PsbS conformations (inset in Figure 2) clearly shows the position of H2 below the membrane plane at neutral pH. Presumably, the deprotonation of Glu 173, which is part of the H2 helix, triggers the exposure of the—now negatively charged—H2 to the aqueous environment. When neutralized, the H2 switches to the hydrophobic environment offered by the membrane (Figure 2B,D). The same behavior is observed in both replicas. However, it must be noted that while for one monomer there is a clear movement of H2 from the membrane to the aqueous phase where it is stable for long time windows, the H2 belonging to the other monomer switches between the aqueous and membrane environment during the simulation time of 400 ns. Nevertheless, the peaks of the tilt angle distributions are shifted to lower values in all the simulations at neutral pH conditions. Our findings are fully supported by the experimental data of FTIR performed at the pH conditions of 5 and 7.5 on both the PsbS wild type (WT) and Glu173Gln mutant.^23^ Here, the Glu 173 is replaced by a glutamine residue to mimic the behavior of an always protonated glutamate. The mutant always assumes the low pH conformation where H2 is in the hydrophobic environment.

**Figure 2:**
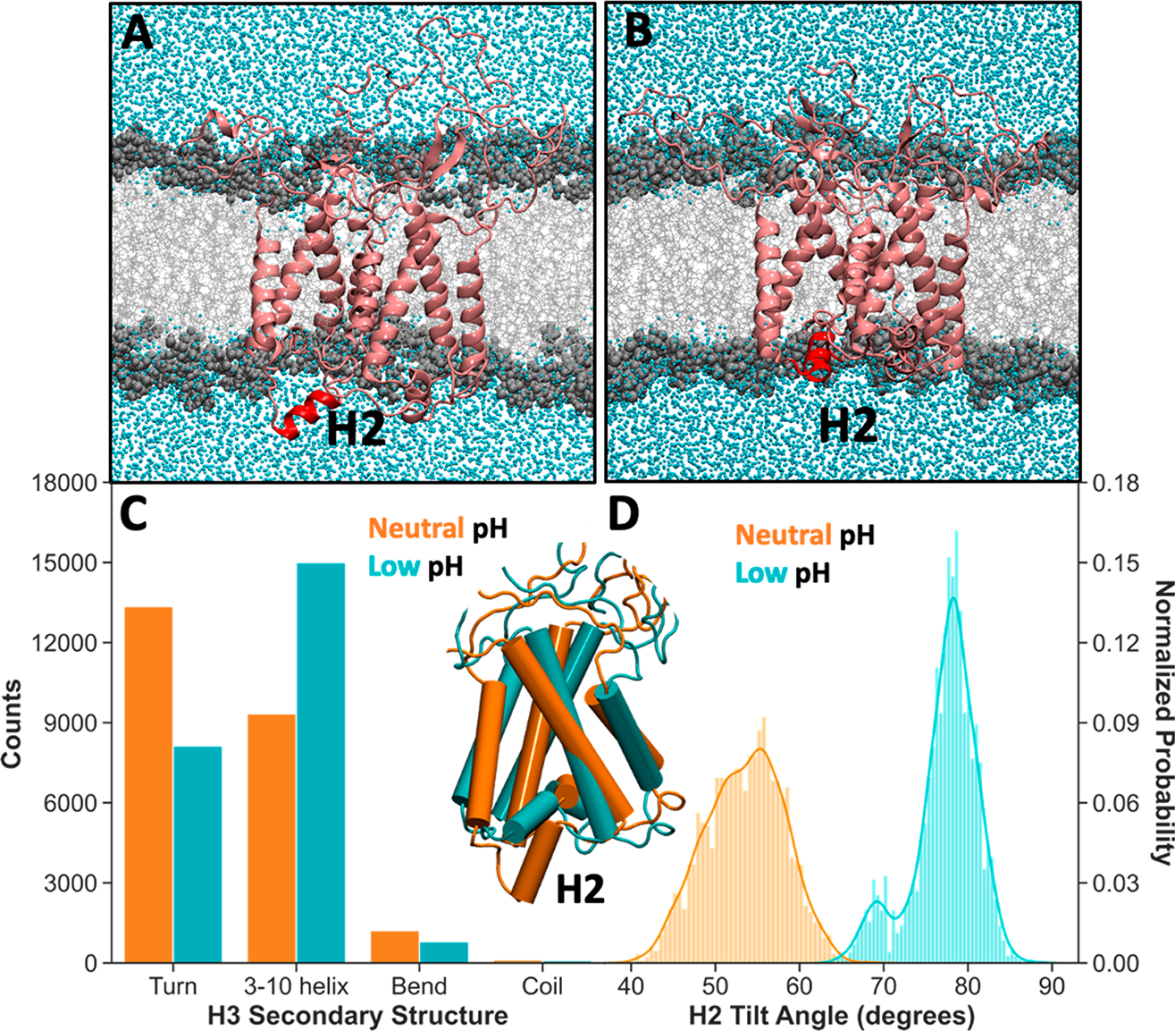
Structural analysis of PsbS dimer highlighting the shift of H2 into the aqueous phase at neutral pH and the folding of H3 into 3_10_ helix at low pH. (A, B) Snapshots of the all-atom simulations exhibiting two distinct protonation layouts. All the Glu residues are deprotonated (A) in the atomistic structure restored from the CG simulation at pH 7. Here the H2 (highlighted in red) moves from the membrane to the aqueous environment. The Glu residues with a significant p*K*_a_ shifts (red residues in Figure 1B) are protonated in the atomistic structure (B) backmapped from the CG simulation at pH 5. (C) Counting plot of the secondary structure elements of H3 from neutral (orange) and low (cyan) pH simulations. At low pH H3 is folded into a 3_10_ helix for most of the simulation time. (D) Distribution of H2 tilt angle computed for the atomistic simulations mimicking neutral (orange) and low pH conditions (cyan). The shift of peak of the H2 tilt angle distribution indicates the movement of H2 from the membrane to the aqueous phase. The overlap of representative structures of both the atomistic simulations clearly shows the position of H2 below the membrane plane.

The other protein portion, sensitive to the pH variation, is the small H3, which has been predicted to undergo a secondary structure change from the turn state at neutral pH to a 3_10_ helix at low pH.^24^ The analysis of the H3 secondary structure including the behavior per residue over the time and the counting of secondary structure elements for both pH conditions is shown in Figure S8 for both the simulations, while the total secondary structure counting plot as average over all the simulations and all the residues is presented in Figure 2C. On average, at neutral pH, H3 is in its disordered turn state while at low pH it is folded into a 3_10_ helix for most of the simulation time. However, this helical portion exhibits high conformational disorder, switching continuously between a 3_10_ helix and turn state (see Figure S8, right panels). Interestingly, in the Glu173Gln mutant^23^ H3 keeps its 3_10_ conformation even at neutral pH, presumably because the H2 partner remains in the membrane available for H-bond contacts. It turns out that H2 and H3 behave in cooperation, as long as H2 retains its position in the membrane H3 does not completely unfold, because of the favorable contact at the dimer interface.

The previous constant pH simulation does not predict the passage of H2 into solution at neutral pH.^24^ The movement of H2 requires and/or is coupled to a protein conformation not covered by these short simulations. The conformational space sampled by the microseconds long CG simulation, as depicted by the pairwise RMSD (Figures S9 and S10), includes mainly the rearrangement of the transmembrane helices TM3 and TM4. Because H2 is connected through loop regions to both of these transmembrane helices, that could allow the spontaneous movement of H2 when the atomistic resolution is restored. It is worth noting that both the new open arrangement of TM3 and TM4 and the motion of H2 in PsbS appears similar to the conformational change of the LHCII minor antenna CP29,^38−40^ where a lumenal “open” conformation obtained through an enhanced sampling approach exhibits a larger interhelical angle between TM helices A and B (corresponding to TM3/TM4) and more lumen-oriented conformation of helix D (corresponding to H2).^39^

To the best of our knowledge, our results provide the first atomistic model of PsbS with H2 in solution, i.e., the protein conformation in its dimeric state at neutral pH. A change in pH thus affects the conformations of both H2 and H3, which lie at the dimeric interface (see Figures 1 and 2). In the low pH conformation, H2 is in the membrane environment and H3 is folded into a 3_10_ helix, allowing the formation of direct contacts between them in the dimeric state. At neutral pH, the H2 goes in solution, H3 forms a loop, and there is no possibility for direct interaction, offering a potential route for tuning the relative stability of PsbS homodimers versus the heterodimers with other LHC partners. Because the PsbS in its monomeric form is supposed to interact with other protein partners during NPQ, it is worth to investigate how the PsbS conformations and the protonation states of the titratable sites affect the stability of the dimer. An unbiased simulation of a protein–protein dissociation event is challenging even at CG resolution. Indeed, we do not detect any spontaneous monomer formation in the CG simulation of both the low and neutral pH conformations. We, therefore, coupled the CG model with an enhanced sampling technique (i.e., metadynamics)^30^ choosing as collective variable (CV) the center of mass between the monomeric subunits of PsbS.^41,42^ The computational details of the metadynamics simulations, including evidence for convergency (Figures S11 and S12) are given in the Supporting Information. To dissect the effects that the different protonation states and conformations have on the dissociation energy we consider two systems, shown in Figure 3: the conformation at neutral pH, where H2 is in the aqueous environment and all Glu residues deprotonated and a conformation at low pH, where H2 is in the membrane. For the low pH case, we consider two protonation layouts: most of the Glu (12 out of 15) are protonated in the Low pH-A conformation (Figure 3), according to the p*K*_a_ shift computed in the constant pH simulation. However, because the acidification necessary for the NPQ activation takes place only on the lumenal space of the native thylakoid membrane, the titratable residues close to the stromal region could be unaffected by the pH variation. Therefore, we consider only the Glu exposed to the lumen to be protonated in the Low pH-B conformation, which would correspond to the protonation state inside the thylakoid membrane upon acidification of the lumenal side.

**Figure 3:**
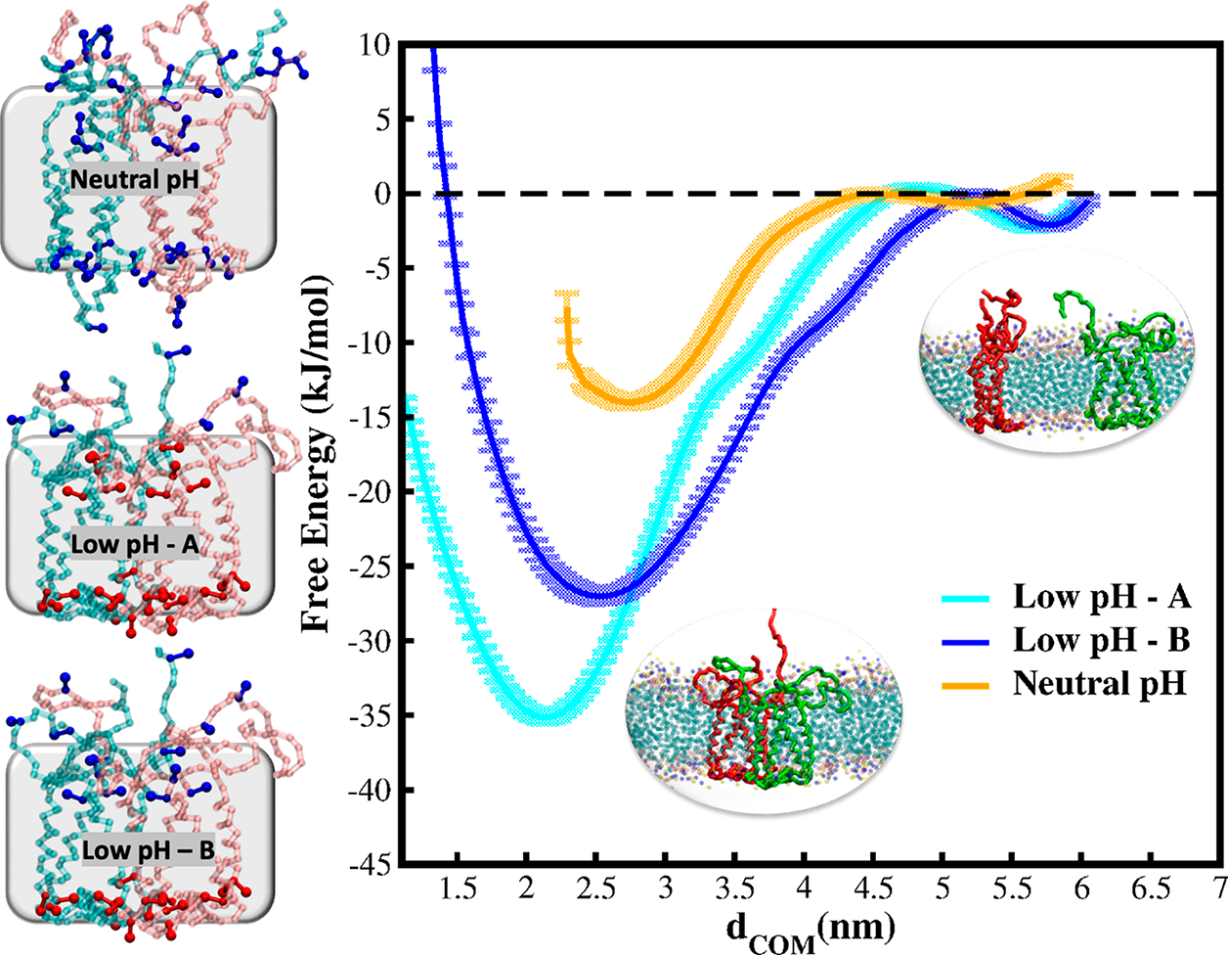
CG metadynamics simulations for the dissociation of the PsbS dimer. Left panel: CG configurations used for the simulation of the dissociation process. Neutral pH conformation (right panel) exhibits H2 in the aqueous phase, and all the Glu are deprotonated (highlighted as blue residues in the ball-and-stick representation); in the low pH conformation H2 is in the membrane phase, and two protonation layouts are considered: most of the Glu (Low pH-A) and the Glu residues exposed to lumen (Low pH-B) are protonated (highlighted in red). Right panel: free energy profiles (kJ/mol) along the CV (nm) for the low and neutral pH conformations. Neutral pH conformation has the lowest energy (∼15 kJ/mol) for the dissociation of the PsbS monomeric subunits (orange line). Both the low pH conformations have dissociation energies of ∼30–35 kJ/mol (cyan and blue lines).

We report the dimerization free energy profiles for the two systems as a function of the CV (*d*_COM_) in Figure 3. In all three cases, the free energy minimum corresponds to the dimeric state. However, the barriers for the dimer dissociation are significantly different and follow a clear trend. The dissociation of the neutral pH dimer requires the lowest energy (15 kJ/mol), while both the free energy profiles for the low pH dimer conformation converge to around 30–35 kJ/mol. The free energy minima are localized between 2.2 and 2.5 nm (*d*_COM_ from the unbiased simulations is reported in Figure S13). Because of the electrostatic repulsion of the deprotonated Glu at the dimeric interface, the free energy minimum of the Low pH-B conformation is localized to larger values of the *d*_COM_ with respect to the Low pH-A conformation. Given the structural differences between low and neutral pH PsbS dimeric forms, the main contribution to the dimer stability arises from the interaction between H2 and H3. At neutral pH, when H2 is in solution, the network of lumenal H-bonds at the dimeric interface is weakened compared to the PsbS conformation where H2 lies in membrane phase (Figure S2). This is also supported by the findings of Fan et al.,^22^ where cross-linking experiments between the PsbS monomers suggest that PsbS adopts a different conformation at neutral pH with a larger distance at the lumen side between the two monomeric subunits.

When the pH drops to 5, the H2 switches to the membrane phase and Glu173 interacts via H-bond with Ile 74 and Tyr 75 (Figure S2), contributing to the dimer stability. Note that the dissociation barrier at neutral pH is only a few *kT* (∼15 kJ/mol), implying that an equilibrium could exist between the monomeric and dimeric forms. Moreover, the presence of the characteristic lipids of the native thylakoid membrane could modulate this equilibrium, further facilitating the monomers dissociation. In the context of PsbS involvement in NPQ, the distinction that is usually made is between PsbS *inactive* and *active* forms, referring to the neutral and low pH conformations, respectively. According to our results, in the inactive state, the two monomers weakly interact while in the active state, the H2 position in membrane and the H3 folding gives a certain stability to the dimeric structure. Whether a dimeric PsbS is also dominant under NPQ conditions is rather questionable. In our simulation, only the interactions between PsbS monomer subunits are taken into account. However, a popular model for the NPQ involves a pH-dependent docking mechanism between the PsbS monomer and LHCII trimer.^10,16,17,43^ It has been shown through structural alignment that any high-resolution structure of the homologous LHCs,^44,45^ which has a folding similar to PsbS, can form a heterodimeric structure in partnership with the active form of the PsbS monomer, restoring the interactions between the amphipathic helices at the dimeric interface.^24^ The dissociation of the PsbS dimer takes place together with a change of localization within the PSII supercomplex; it was observed that the dimers are mainly associated with the PSII core, while monomers with the LHCs.^5,10^ Moreover, despite PsbS not being a cofactor binding subunit, it operates together with zeaxanthin in inducing an efficient quenching.^46^ The presence of zeaxanthin further affects the affinity of the PsbS with LHCII subunits, enhancing the interaction with minor antenna complexes.^47,48^

On the basis of all of this data, we conjecture that the *inactive* neutral pH conformation of PsbS, with H2 in solution, lacks the structural element that allows a stable binding to another protein partner, as shown by the dissociation free energies of the PsbS dimers. Under these conditions, an equilibrium between PsbS dimers (possible associated with the PSII core) and monomers exists. Whereas, in the *active* low pH conformation, where the H2 resides in the membrane phase and is available as H-bond copartner, the PsbS monomers will have an enhanced interaction with the LHC subunit. This type of pH-dependent docking is at the basis of NPQ.

In conclusion, with our results we find a good match with experimental data regarding the structural response of PsbS to the pH. The constant pH method within the Martini framework has been applied here for the first time to a protein system and,after backmapping, provided a detailed atomistic model of the PsbS dimer structure at neutral pH, including the solvent-exposed H2 helix. These structural details together with metadynamics simulations allowed us to understand how the different PsbS conformations/protonation states affect the free energies of dissociation. Future work should include the other PsbS putative binding partners (i.e LHCs), aimed at characterizing the specific interactions locking the PsbS to the LHCs in the NPQ mechanism.

## Supporting Information Available

Computational details for the system setup, all-atom MD, standard and constant pH CG Martini simulations and CG MTD simulations; schematic overview of the simulation workflow (Figure S1), structural analysis of the MD and CG simulation (Figures S2 and S3); schematic representation of Glu titratable bead with the model system titration curve (Figure S4); titration curves for Glu residues not shown in the main text (Figure S5) and protonation state of Glu residues over the time (Figure S6), secondary structure analysis of the all-atom simulations and analysis of tilt angle (Figures S7 and S8); pairwise RMSD and clustering of the constant pH CG simulation (Figures S9 and S10); convergence of the free energy profiles for MTD simulations presented in this work (Figures S11 and S12), full list of the performed simulations (Table S1) and p*K*_a_ values (Table S2) (PDF)
