# Supplementary material for: pH-Dependent Conformational Switch Impacts Stability of the PsbS Dimer
Source: J Phys Chem Lett. 2023 Jan 20;14(4):905–11. doi: 10.1021/acs.jpclett.2c03760 (PMC9900633; doi:10.1021/acs.jpclett.2c03760)
Supplement: Supplementary file 1 — jz2c03760_si_001.pdf [file jz2c03760_si_001.pdf]

Supporting Information

pH-Dependent Conformational Switch Impacts Stability of the PsbS Dimer

Maria Gabriella Chiariello[^], Fabian Grünewald[^], Rubi Zarmiento-Garcia[^], Siewert J. Marrink [^*]

^ Groningen Biomolecular Sciences and Biotechnology Institute (GBB), University of Groningen, Nijenborgh 4, 9747 AG, Groningen, the Netherlands.

* Corresponding author

s.j.marrink@rug.nl

Computational details

In the following sections we will describe the computational details of the presented simulations. A schematic overview is also offered in Figure S1 and Table S1.

1. System setup

The crystal structure of the homodimeric protein PsbS (PDB ID: 4RI2, resolution 3.00 Å) was used for the initial starting configuration. The X-ray structure lacks a number of aminoacids, including the termini and a portion of the stromal loop (residues 108-133). The missing protein domains were reconstructed via homology modelling using the MODELLER software.[1] All the Glu residues were considered de-protonated. The protonation states of titratable residues are the same in both subunits of the PsbS homodimer. The protein was embedded in 1-palmitoyl-2-oleoyl-sn-glycero-3-phosphocholine (POPC) lipid bilayer using the CHARMM-GUI web server.[2] The system was solvated with ~ 34,000 water molecules with a 150 mM NaCl concentration. The simulation box contained ~ 150,000 atoms (L_x = 100 Å, L_y = 100 Å, and L_z = 130 Å). All-atom and coarse-grained (CG) simulations were performed using the GROMACS-2020 package.[3]

2. All-atom MD simulation

Proteins, lipids, ions, and water were described with CHARMM-CMAP [4], CHARMM36 [5], and TIP3P [6] force fields, respectively. Long-range interactions were evaluated using particle-mesh Ewald summation [7] with a 12-Å cut-off in real space. Lennard–Jones (LJ) interactions were truncated at 12 Å with an atom-based force switching function, which starts to be effective at 10 Å. The integration time step was set at 2 fs. Bonds involving hydrogen atoms were constrained using the LINCS algorithm.[8] A Nosé–Hoover chain thermostat[9] was used to maintain the temperature at 298 K. A semi-isotropic Parrinello–Rahman barostat [10] with a reference pressure of 1 atm and isothermal compressibility of 4.5×10^{-5} bar^{-1} was used to maintain the pressure of the system. After equilibration, the system was simulated for 300 ns (Figure S2).

3. Coarse-grained Martini simulations

The Martini 3 force field was used for all the CG simulations.[11] The CG structure of the PsbS protein was generated using the *martinize2*[12] and was embedded into a POPC bilayer, solvated with ~ 14000 water beads with a 150 mM NaCl concentration using the *insane* script.[13] The simulation box contained ~ 22,000 atoms (L_x = 180 Å, L_y = 100 Å, and L_z = 140 Å). Both the elastic network (EN) and Go-like model (Go) were used as additional potentials to preserve the secondary structure domains of the PsbS protein[14] [15] (Figure S3). The EN model adds harmonic potentials between backbone beads with a force constant of 500 kJ mol^{-1} nm^{-2}. The lower and upper elastic bond cut-offs are 0.5 and 0.9 nm. The Go model instead uses LJ potentials defined

between virtual sites located at the position of the backbone beads. The dissociation energy of the LJ interaction is 12 kJ/mol. After equilibration, the system was simulated for 10 μs using a time step of 20 fs. The temperature and pressure were maintained constant at 298 K and 1.0 bar through a velocity rescale thermostat[16] (time constant 1.0 ps) and semi-isotropic Parrinello-Rahman barostat[10] (time constant 12 ps). Electrostatic interactions were treated using the reaction field approach (cut-off at 1.1 nm) and a shifted Van der Waals potential, cut-off at 1.1 nm with the Verlet cut-off scheme.[17]

4. Constant pH CG Martini simulations

We used a recently proposed approach to take into account the pH effect into CG simulations with the Martini 3 force field, denoted Martini sour.[18] In Martini sour, new bead types are introduced: titratable beads and a proton bead. The titratable beads are divided into acids and bases which have an internal geometry (see Figure S4A) that allows to reversibly bind proton beads. The bead representing water is also titratable. The strength of the interaction between water and proton beads is tuned by a LJ potential. In particular, the sigma and epsilon parameters of LJ interaction between water and proton beads are parametrized according to a pH scale of 3-8. For instance, the interaction is weaker at low pH and there is more 'availability' of protons for titratable sites other than water. The interaction between the proton bead and titratable bead is parametrized to reproduce the target pKa of the chemical fragment that the titratable site represents. The proton bead interacts only with water and titratable beads. The method is fully compatible with the Martini framework, with no need to recalibrate the interactions involving neutral beads. The interactions between charged beads are however readjusted since the titratable water model uses a relative dielectric screening constant (epsilon_r =6), different from the standard water which uses epsilon_r =15. In the original work, the free energies of transfer from hexadecane to water (titratable) of a series of titratable compounds have been computed as a function of the pH, providing a good match with experimental values.

During the titration, the degree of deprotonation is calculated through the counting of proton beads bonded to the titratable site at each value of the pH. The titration curves are obtained using the Hill equation, i.e. by means of fitting the resulting points using the equation:

Eq. 1 $$\alpha = \frac{1}{10^{q(pH-pKa)}+1}$$

where α is the degree of deprotonation, q is the Hill coefficient and pKa corresponds to the inflection point of the sigmoid function, here pH = pKa. The scripts to perform the analysis are available at *https://github.com/fgrunewald/titratable_martini_tools* and *http://cgmartini.nl).*

To compute the reference pKa value of Glu in water, we used a pentapeptide composed of an alanine chain with a glutamate residue in the middle. This pentapeptide provides a model for the calculation of pKa values of ionizable aminoacids in water, also for experimental measurements.[19] The CG model is illustrated in picture S4. The side chain of the Glu was

replaced by an acid titratable bead (type P2_4.8)[18] which is able to bind a proton bead. The system is embedded in a box (40 x 40 x 40 Å) of 690 titratable Martini water beads. The simulation was run for 10 ns for each pH values in the 3.0-8.0 range. The titration curve is also shown in Figure S4. The computed pKa value (4.5) is in nice agreement with the experimental value (4.3).[20] We use this curve as reference to quantify the pKa shift of the Glu residues in the PsbS protein, as shown in Figure 1.

To perform the titration of the PsbS dimer, we started from the CG equilibrated structure of PsbS embedded in a POPC bilayer and solvated with water. 15 Glu residues per monomer were replaced with their titratable version, while the standard Martini water was replaced with titratable water. Before the production run the system was minimized and equilibrated for 2 ns at each pH value. The PsbS titration was run for 1 μs at each value of pH in the 3-8 range. All the titrations were carried out using the stochastic dynamics integrator[21] with a time step of 10 fs in an NPT ensemble, where temperature and pressure were maintained constant at 298 K and 1.0 bar through a velocity rescale thermostat (time constant 1.0 ps) and isotropic Parrinello-Rahman barostat (time constant 3 ps). Note, using a semi-isotropic barostat in combination with the titratable water model can result in artificially large box deformations and was therefore avoided. Both the EN and Go potential were employed, providing consistent pKa values. Other settings were the same as described above for the non-titratable CG simulations. We used the same simulation parameters to perform the titration of the alanine pentapeptide as for the titration of the PsbS dimer.

5. Backmapping to all-atom resolution

The configurations from the titratable CG simulations at pH 5 and 7 were converted into all-atom structures through a back-mapping procedure that makes use of the *backward* script.[22] To mimic the effect of the pH, we back-mapped into atomistic models where the protonation state of the Glu residues is settled according to the pKa shifts predicted with the titratable CG model. In particular, the CG structure resulting from the constant pH simulation at pH 5 was back-mapped into an atomistic model where 12 over 15 Glu are protonated. On the other hand, the CG structure resulting from the constant pH simulation at pH 7 was back-mapped into an atomistic model in which all Glu are de-protonated. We extracted two configurations (here called Trajectory I and II) for each pH value (7 and 5) and all the simulations were run for 400 ns using CHARMM force field, as for the initial all-atom simulation. Other settings are the same as described above in section 2. Before the production run, the two systems were minimized (two cycles of 500 steps energy minimization) and equilibrated (four cycles of 1 ns position restrained NVT simulations with increasing time step of 0.2,0.5,1 and 2 fs).[22] Only the 400 ns long production trajectories were used for the analysis presented in this work.

6. Coarse-grained Martini coupled with metadynamics

In order to simulate the dimer dissociation process, we employed metadynamics in its well-tempered version (WT-MTD).[23] As explained in the main text, we considered three systems: the low and neutral pH configurations where the H2 is in membrane and aqueous environment, respectively (see Figure 2). In addition, different protonation layouts were used for the low pH configuration (labelled as low pH A and B) (Figure 3). The starting low and neutral pH configurations were created by transforming the respective atomistic structures (described in section 5) into their corresponding CG Martini representation. The atomistic configuration extracted from the all-atom simulation at neutral pH and used for the CG mapping, exhibits H2 of both monomers in aqueous environment. The protonation state of Glu residues is mimicked through the employment of charged and neutral beads, in particular: the side chain of the Glu residues are described by means of charged beads in the neutral pH form, while neutral beads are instead employed for most of the Glu residues in both the low pH conformations. Before the MTD, the CG model of the low and neutral pH configurations were simulated for 15 μs; here we did not observe any spontaneous monomer formation. The EN potential was added to maintain the secondary structure. The WT-MTD simulations were carried out using the same setup as the unbiased CGMD simulation based on the standard Martini 3 force field, with the following parameters for the deposited bias: Gaussian height 1.2 kJ/mol, Gaussian sigma 0.1 Å, bias factor 12, and Gaussian deposition rate of 100 ps. We defined the distance between the centres of mass of two monomeric sub-units (d_{COM}) as collective variable (CV). The aim of the MTD simulations is to compare free energy profiles for the dissociation of the neutral and low pH conformations which differ mainly for the position of the small amphipathic helices (H2 and H3) at the dimeric interface. Therefore to keep the native relative orientation of the two monomers during the sampling, we used restraints on two rotational angles between the transmembrane helices as described in Johnston et al.[24] We first determined the values of the torsional angles in the native configurations from the unbiased simulations. These restraints, with potential function U, are activated when the angles move away from the equilibrium values. In particular, U reads:

$$U = k/2\ (x_i - a_i)^2$$

Where k= 100 kJ mol^{-1} $degrees^{-2}$, a_i is the threshold value. To quantify the effect of the potential, one can consider that the energy penalty for a deviation of 0.1 degree is 0.5 kJ/mol, which is small enough to allow oscillation of the angles and the exploration of slightly different configurations. The bias due to the harmonic restraint was monitored during the simulation and was included in the final reweighing procedure to reconstruct the free energy landscape using the algorithm described in Ref. [25]

The simulations were run for a time ranging from 41 to 46 μs for the three systems and convergence was assessed by checking the diffusive behaviour of the collective variable, the time evolution of the free energies profiles along the CV (Figure S11) and the difference in the free energies between minimum and maximum values of the PMF over the simulation time (Figure S12). The error bars were calculated using the block analysis.

The WT-MTD simulations were performed using the GROMACS-2020 package [3] patched with the PLUMED 2.5 plugin. [26] [27]

Supporting Figures

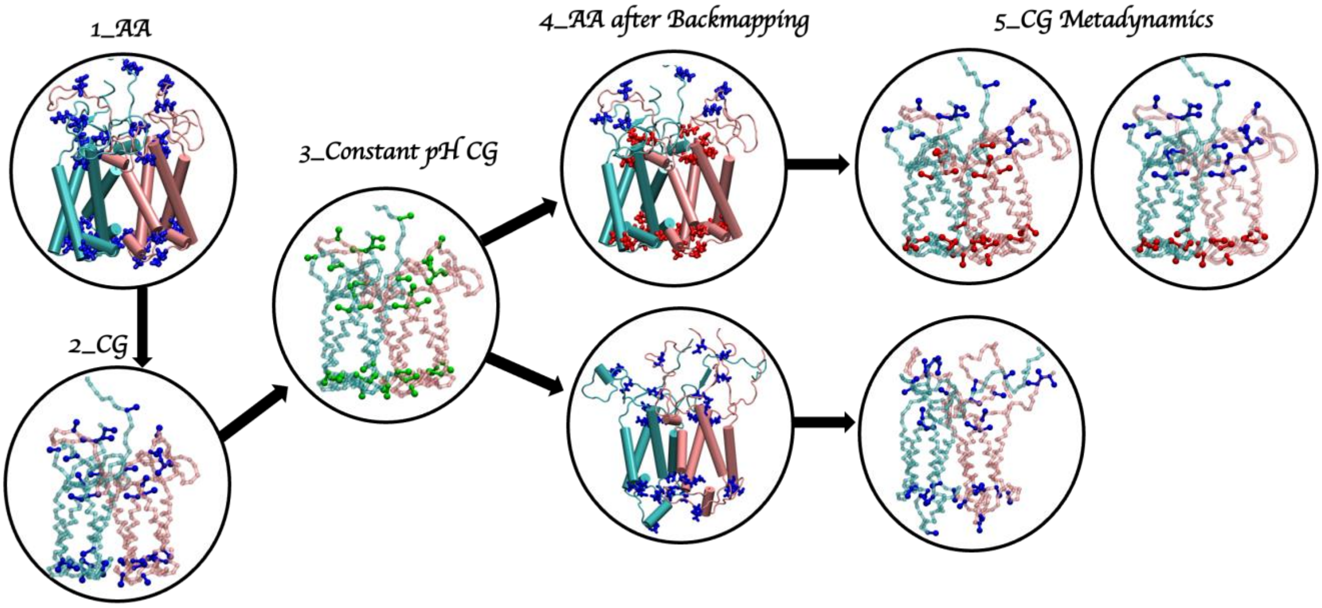

Figure S1 Schematic representation of the workflow for the all-atom (AA) and coarse-grained (CG) simulations of the PsbS dimer. The Glu residues are colored according to their protonation state: de-protonated (blue), protonated (red), or described by means of titratable beads (green). The POPC bilayer is omitted for clarity. The X-ray structure of the PsbS is used as starting point for the AA simulation (**1_AA**), an AA representative snapshot is converted into a CG model (**2_CG**). Then we perform the constant pH CG simulation (**3_Constant pH CG**), by converting the Glu beads into titratable sites. The CG structures are backmapped into two atomistic models with different Glu protonation states to mimic the neutral and acid pH conditions (**4_AA after Backmapping**). Two configurations (called Trajectory I and II in the following text) per each pH values are extracted and simulated with atomistic resolution. Finally, the neutral and low pH atomistic conformations are converted again into CG models to perform the metadynamics simulations (**5_CG Metadynamics**). An overview of the performed simulations with further details is also presented in Table S1.

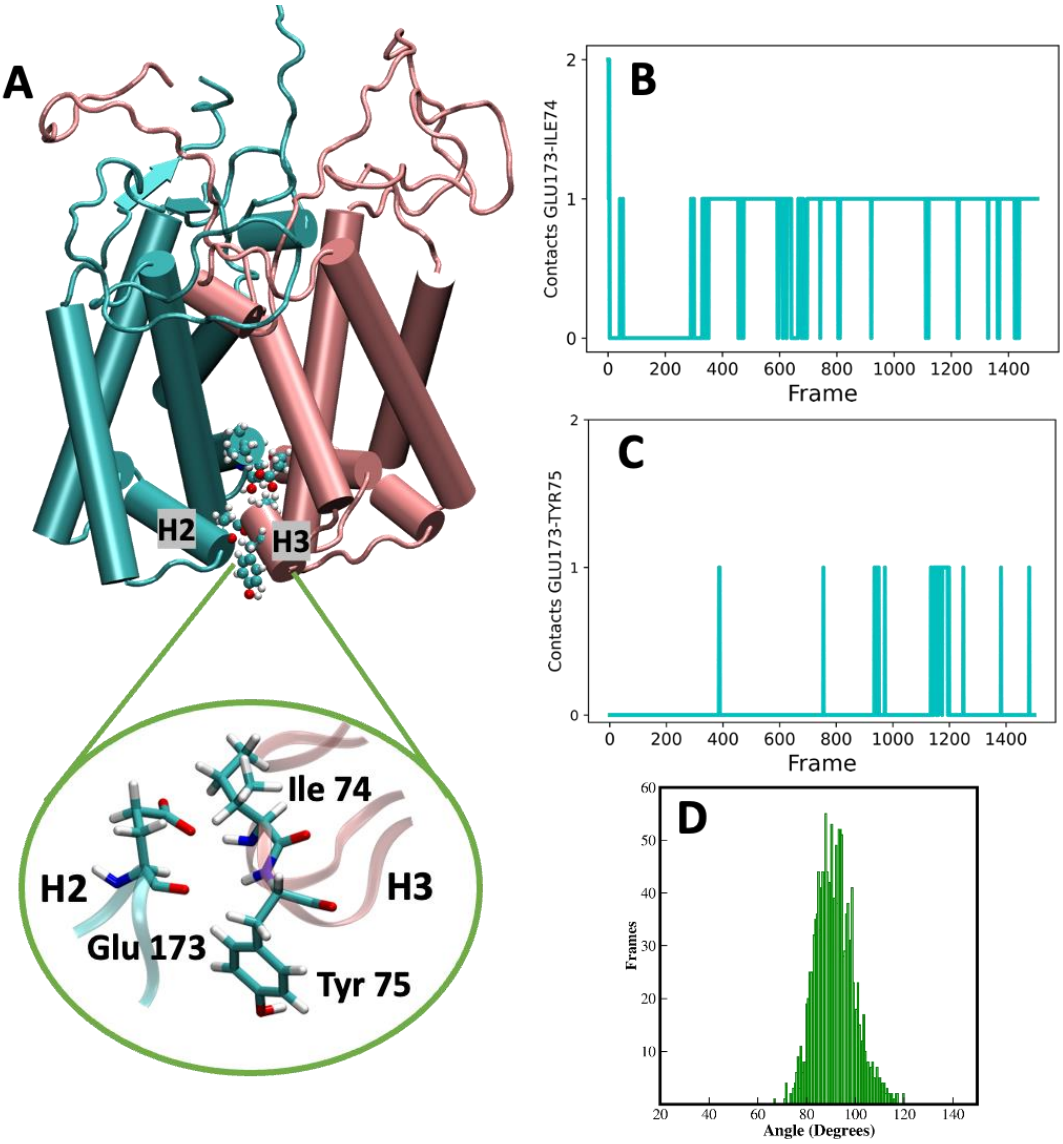

Figure S2. A) Structure of PsbS dimer used as starting configuration for the initial all-atom MD simulation. All the Glu residues are considered to be de-protonated. The short helices H2 and H3 contribute to the dimer stability via the H-bonds between the Glu 173 of H2 and Ile 74 and Tyr 75 of H3 shown in the inset. **B)** and **C)** H-bonds contacts between Glu 173 - Ile 74 and Glu173 - Tyr 75 monitored during the initial all-atom MD simulation. As the PsbS is a homodimer, two H-bond connections between these amino acids can be established at the dimeric interface. **D)** Distribution of the H2 tilt angle sampled during the MD simulation. The average value is around 90 degrees and the helix maintains its position in the membrane environment during the simulation time of 300 ns.

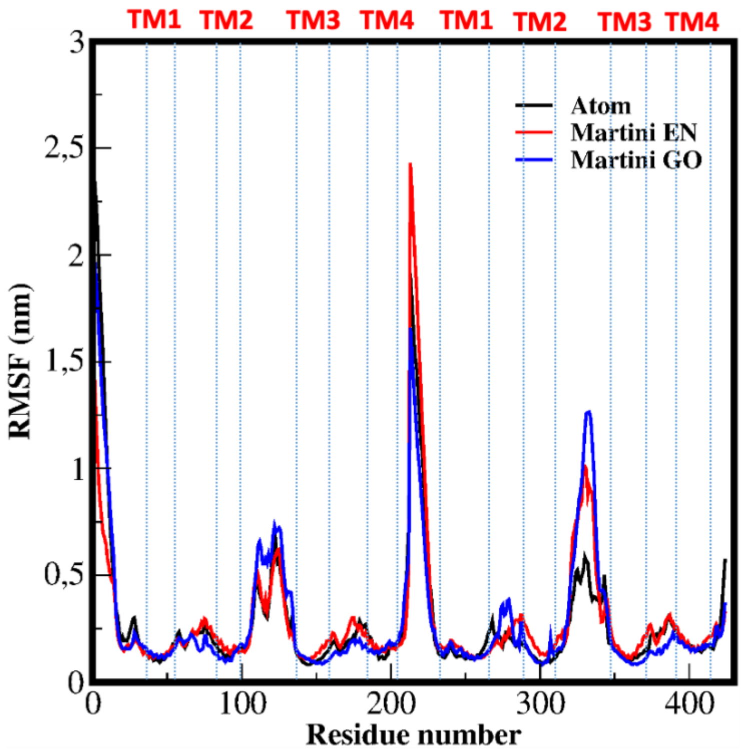

Figure S3. Comparison of the root mean square fluctuations (RMSF) computed for the reference all-atom simulation (black) and optimized Martini model with both EN (red) and Go (blue) additional potentials. The transmembrane domains are highlighted and confined between dashed lines. The protein domains corresponding to the stromal loop (residues 94-137 and 306-349), N terminus (1-25 and 213-237) and C terminus (198-212 and 410-424) show the highest values of the RMSF reflecting the high disorder characterizing these regions.

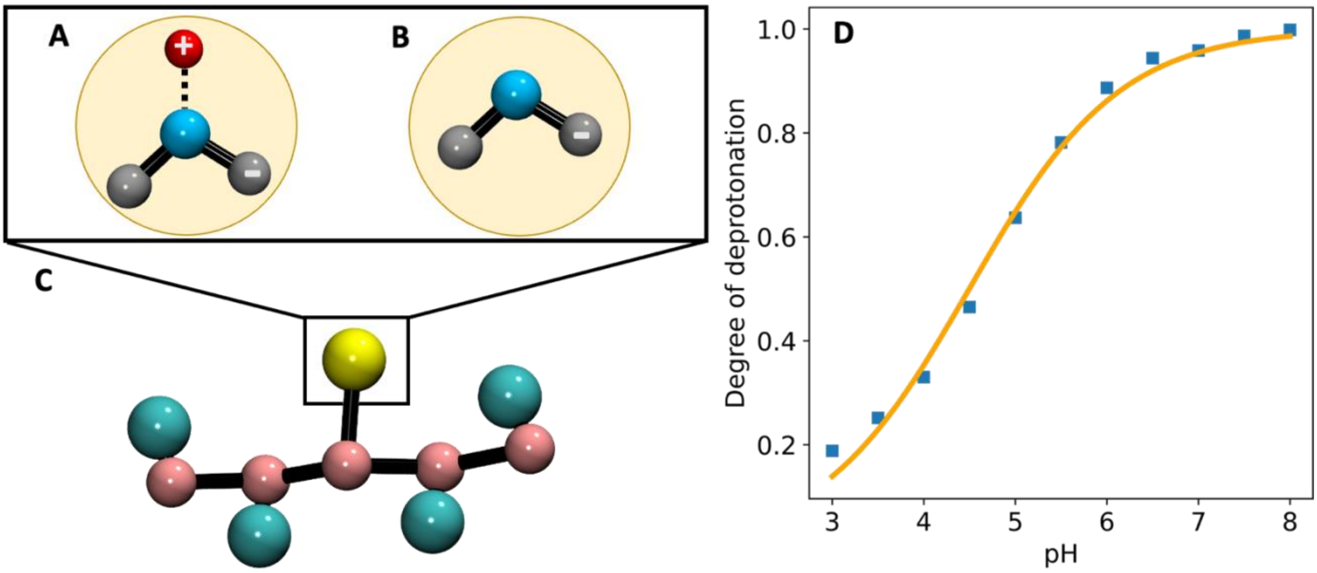

Figure S4. Titratable acid bead in neutral **A)** and ionized **B)** form used to describe the titration of a Glu residue. The bead has a central site (cyan particle) which is able to bind a positively charged proton bead (red particle) and two dummy particles (grey particles) with a negative charge. **C)** CG representation of the alanine pentapeptide (backbone beads in pink, Ala side chain beads in green and Glu side chain in yellow) used for the calculation of the pKa value of Glu in water. **D)** Titration curve of the Glu in the alanine pentapeptide system computed at CG level. The pKa is 4.5, in nice agreement with the experimental value of 4.3.[19]

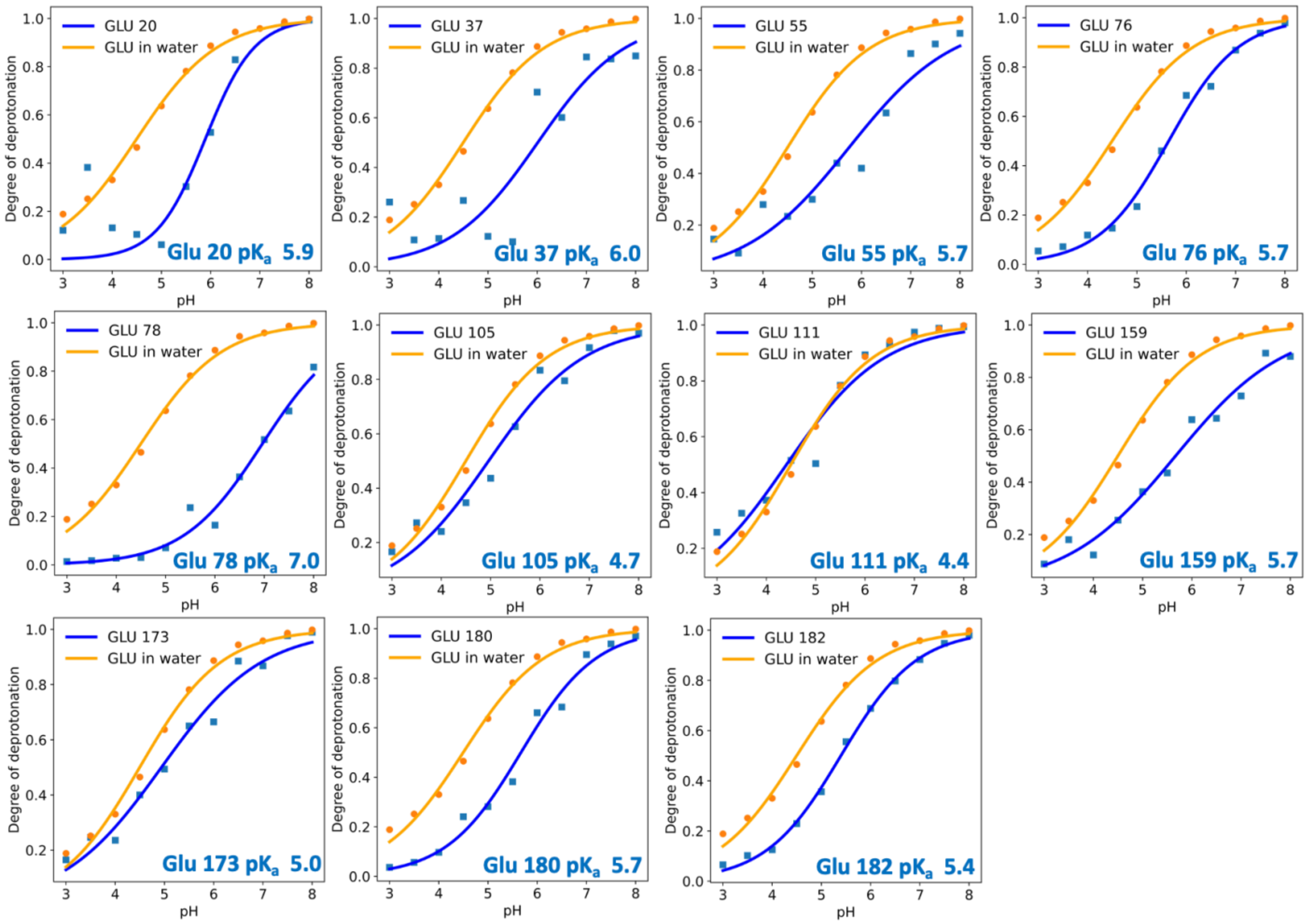

Figure S5. Titration curves of the Glu residues not shown in the main text. The full list of pKa values is reported in Table S2, while the details of the simulation and analysis are discussed in the computational details.

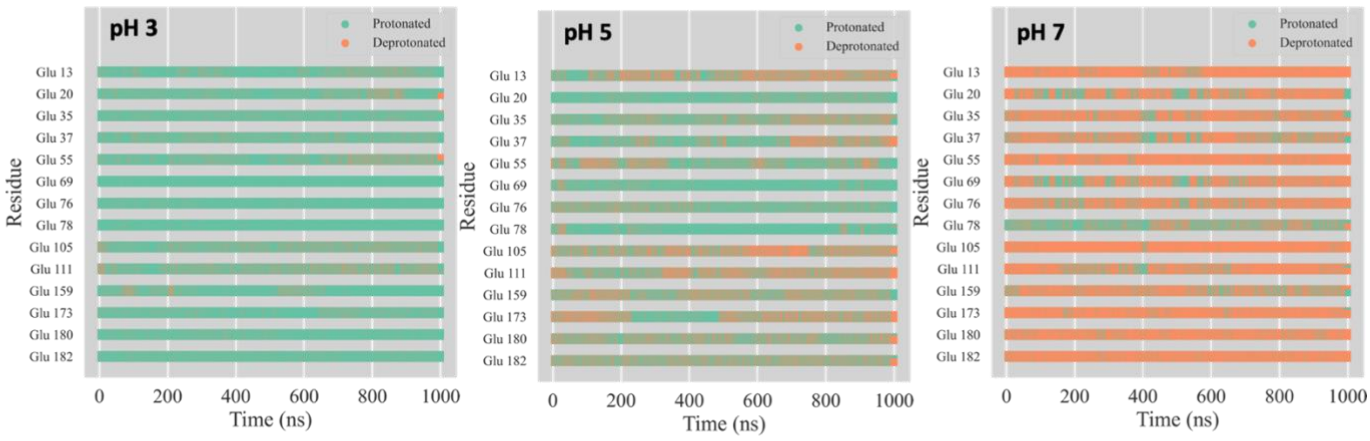

Figure S6. Protonation state over the time of all the Glu residues computed from the constant pH CG simulations at pH 3, 5 and 7.

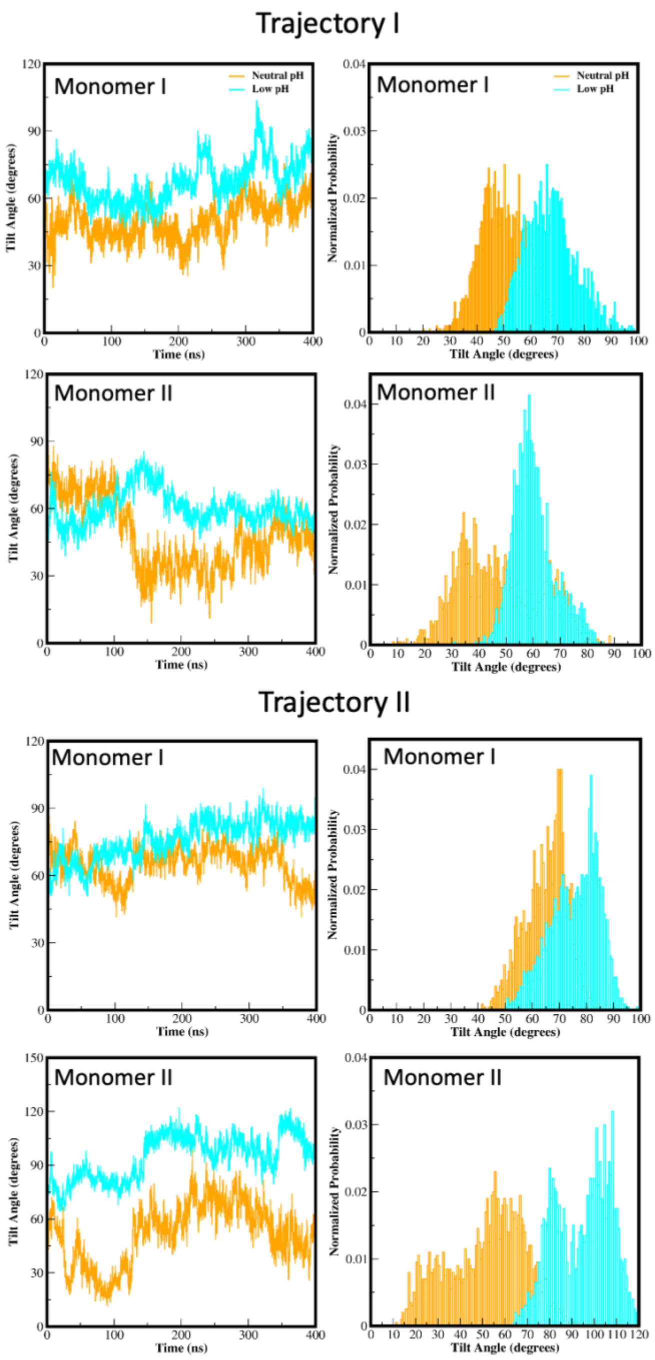

Figure S7. Analysis of the H2 tilt angle computed from the two AA simulations (Trajectory I and II) after backmapping procedure. The behaviour of the H2 tilt angle of both monomers are shown. **Left panels:** Time evolution of the H2 tilt angle at neutral (orange) and low (cyan) pH simulations. The corresponding distributions are reported in the **right panels.** In Figure 2D of the main text, we present the total distribution obtained as average of both monomers and replicas of the low and neutral pH simulations here presented but discarding the first 100 ns, i.e. after the H2 transitions took place.

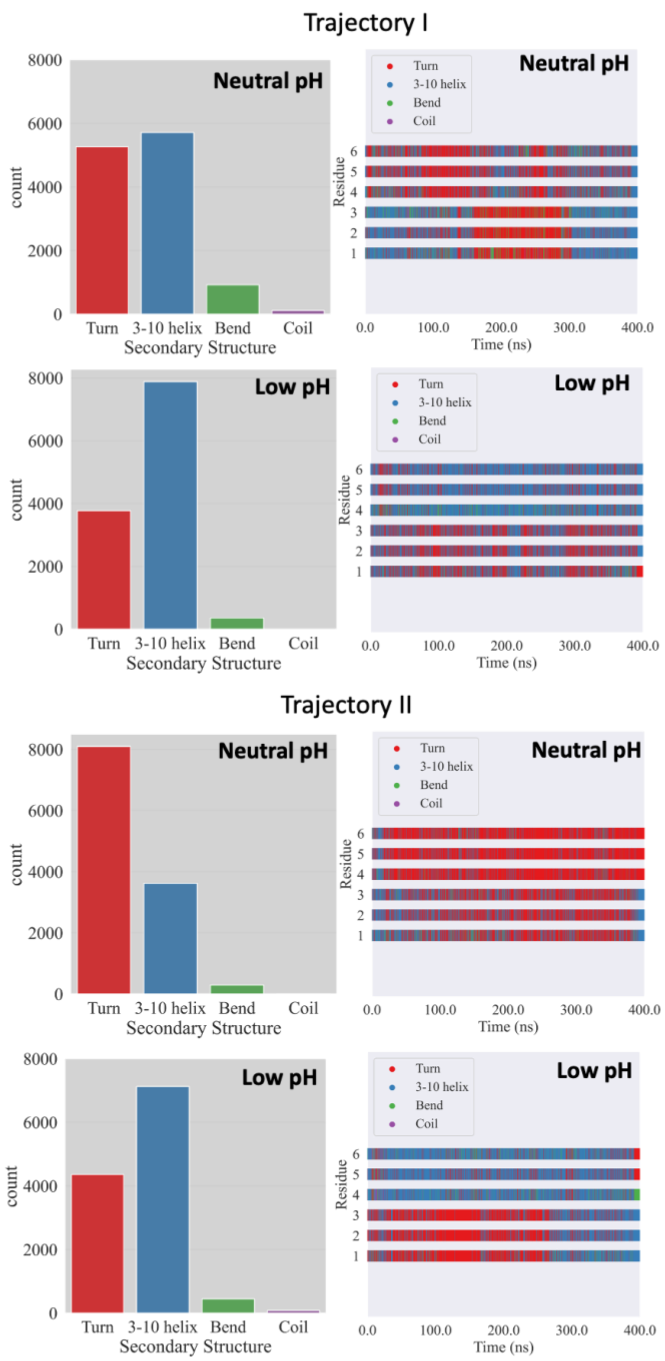

Figure S8. Secondary structure analysis of H3 performed with the dssp Gromacs tool on the two AA simulations (Trajectory I and II) after the backmapping procedure, with all Glu deprotonated or 12 Glu protonated, mimicking the neutral and low pH conditions, respectively. **Left panels:** Counting plot of the secondary structure elements found for H3 at low and neutral pH in both monomers (resid: 74-76 in Monomer I and 286-288 in Monomer II). **Right panels:** time evolution of the secondary structure elements for the six residues of H3. In both the trajectories H3 is folded into 310 helix for most of the simulation time. The total counting plot obtained as average of all neutral and low pH simulations is shown in main text as Figure 2.

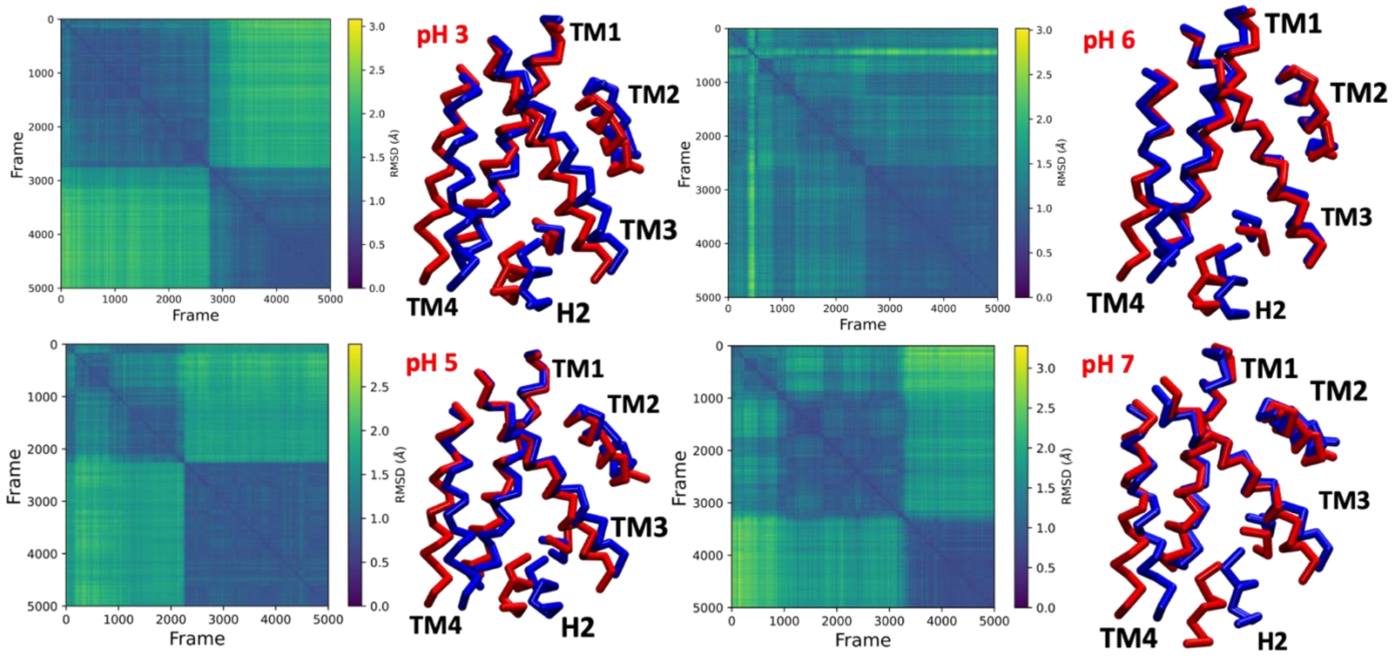

Figure S9. Pairwise RMSD computed for the CG Martini trajectories at pH 3, 5, 6 and 7. It is used to characterize the conformations explored during the CG simulation. The RMSD is computed between every frame of the trajectory. We identify two regions sharing similar RMSD and a conversion between them at frame 2800 (560 ns) at pH 3, 2200 (440 ns) at pH 5, 2700 (540 ns) at pH 6, and 3200 (600 ns) at pH 7. The pairwise RMSD is used as criteria to perform a clustering of the structures, i.e., the frames of the trajectory are grouped based on their similarity. The algorithm used to perform the clustering is described in [28] . Two clusters are identified and their alignment is shown in the **right panels.** The loop domains have not been included in both the calculations of the pairwise RMSD as well as in the clustering procedure. This allows us to focus on the motions and conformations of the transmembrane and amphipathic helices. The main structural difference coming from the clustering is the relative orientation of TM4 and TM3. Both TM4 and TM3 are connected through loop regions to the H2. The two conformations differ mainly for the relative arrangement between TM3 and TM4. H2 follows the movement of TM4, but remains in the membrane environment. This relative rearrangement of the transmembrane helices seems to happen regardless of the pH conditions.

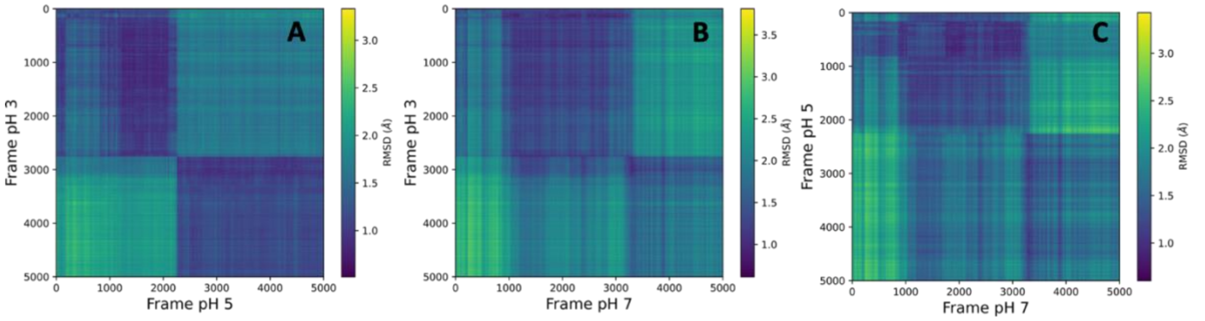

Figure S10. Pairwise RMSD between trajectories at pH 3 and 5 (A), pH 3 and 7 (B) and pH 5 and 7 (C).

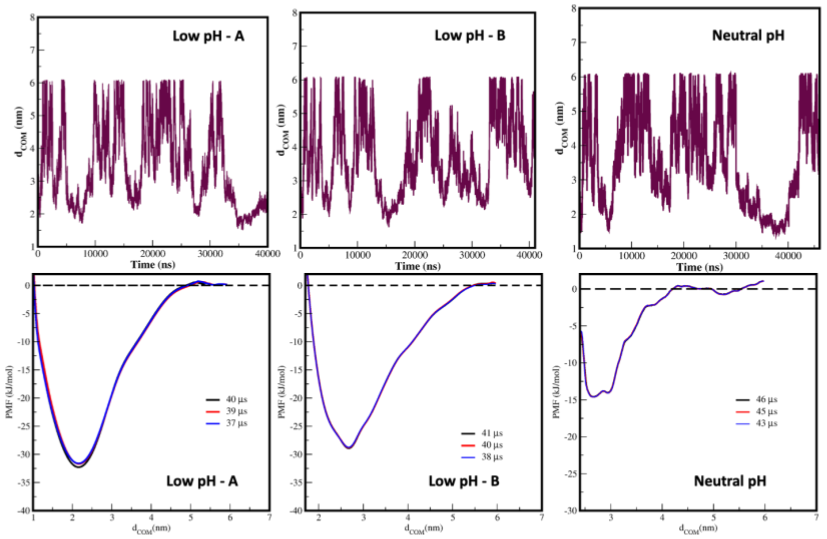

Figure S11. CG MTD simulations. Behaviour of the CV over the simulation time (upper panel) and time evolution of the free energy profiles along the CV (d_{COM}) (lower panel) for the three systems considered: Low pH conformation - A (total simulation time 40 µs), Low pH conformation - B (total simulation time 41 µs) and Neutral pH conformation (total simulation time 46 µs).

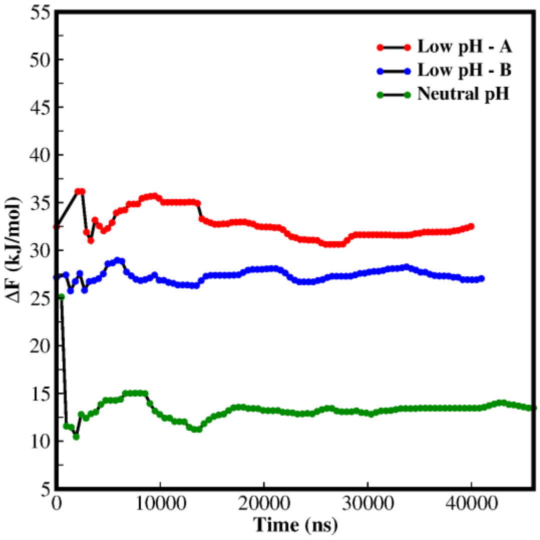

Figure S12. Free Energy difference (ΔF (kJ/mol) computed between the minimum and maximum of the free energy profiles for both Low pH and Neutral pH systems over the simulation time.

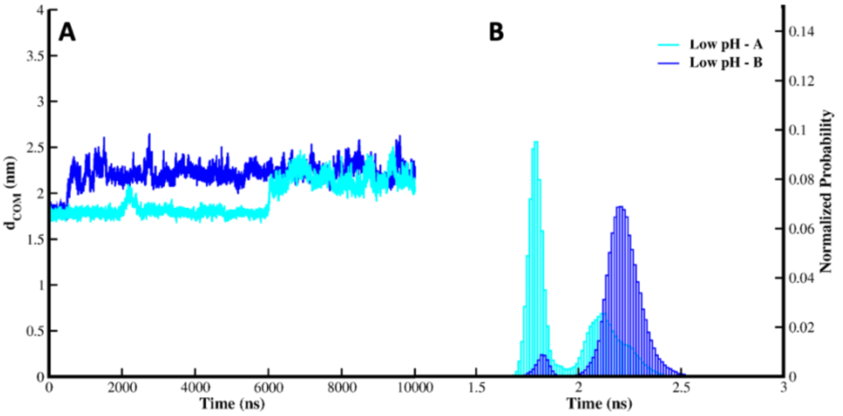

Figure S13. Time evolution of the distance between the center of mass (d_{COM}) of the monomers A) and corresponding probability distributions B) computed from 10 μs unbiased CG simulations for the Low pH – A (cyan) and B (blue) conformations. The systems evolve from the d_{COM} of the crystallographic structure (1.8 nm) to a minimum where d_{COM} oscillates around 2.1 nm (low pH -A) and 2.3 nm (low pH -B).

Supporting Table

Table S1. Full list of the presented simulations with details regarding resolution, employed force field, protonation of the Glu residues and total simulation time (ns). See also Figure S1 for a graphical overview.

	Resolution	Force Field	Protonation State Glu Residues	Enhanced Sampling	Total Time (ns)
**1_AA**	AA	CHARMM	All Glu deprotonated	NO	300
**2_CG**	CG	Martini 3	All Glu deprotonated	NO	1 x 10^4
**3_constant pH CG^**	CG	Titratable Martini 3	Variable	NO	1.1 x 10^5
**4_AA after Backmapping**	**TRAJECTORY I**				
	AA	CHARMM	All Glu deprotonated	NO	400
	AA	CHARMM	24 Glu protonated	NO	400
	**TRAJECTORY II**				
	AA	CHARMM	All Glu deprotonated	NO	400
	AA	CHARMM	24 Glu protonated	NO	400
**5_CG Metadynamics**	CG	Martini 3	All Glu deprotonated	YES	4.6 x 10^5
	CG	Martini 3	24 Glu protonated	YES	4 x 10^5
	CG	Martini 3	16 Glu protonated	YES	4 x 10^5

^ In the constant pH simulation, 11 MD runs of 1 µs covering a pH range of 3-8 are performed.

Table S2. Full list of the computed pKa values and standard error (SE) from the fitting of the titration curves using the Hill equation (Eq. 1). The pKa values of the previous constant pH all atom (AA) simulation are also reported. The Glu residues exposed to the lumen and stromal are coloured in red and blue, respectively. The Glu undergoing a pKa shift > 0.5 with respect to the pKa value of Glu in water (4.5) are highlighted in bold font.

	**pK_a constant pH Martini 3**	**SE**	**pK_a constant pH AA from Ref.** [29]
Glu 13	4.4	0.2	3.9
**Glu 20**	**5.9**	0.3	4.4
**Glu 35**	**5.6**	0.6	4.1
**Glu 37**	**6.0**	0.4	3.5
**Glu 55**	**5.7**	1	-
**Glu 69**	**6.7**	0.7	5.2
**Glu 76**	**5.7**	0.1	5.7
**Glu 78**	**7.0**	0.9	6.3
Glu 105	4.7	0.6	3.4
Glu 111	4.4	0.3	3.9
Glu 141	-	-	5.1
**Glu 159**	**5.7**	0.2	4.1
**Glu 173**	**5.0**	0.3	6.1
**Glu 180**	**5.7**	0.8	6.4
**Glu 182**	**5.4**	0.1	7.7

References

(1) Webb, B.; Sali, A. Comparative Protein Structure Modeling Using MODELLER. *Curr. Protoc. Bioinform.* **2016**, *54*, 5.6.1-5.6.37.
(2) Lee, J.; Cheng, X.; Swails, J. M.; Yeom, M. S.; Eastman, P. K.; Lemkul, J. A.; Wei, S.; Buckner, J.; Jeong, J. C.; Qi, Y.; et al. CHARMM-GUI Input Generator for NAMD, GROMACS, AMBER, OpenMM, and CHARMM/OpenMM Simulations Using the CHARMM36 Additive Force Field. *J. Chem. Theory Comput.* **2016**, *12*, 405-413.
(3) Abraham, M. J.; Murtola, T.; Schulz, R.; Páll, S.; Smith, J. C.; Hess, B.; Lindahl, E. GROMACS: High Performance Molecular Simulations Through Multi-Level Parallelism from Laptops to Supercomputers. *SoftwareX* **2015**, *1-2*, 19-25.
(4) Mackerell Jr, A. D.; Feig, M.; Brooks Iii, C. L. Extending the Treatment of Backbone Energetics in Protein Force Fields: Limitations of Gas-Phase Quantum Mechanics in Reproducing Protein Conformational Distributions in Molecular Dynamics Simulations. *J. Comput. Chem.* **2004**, *25*, 1400-1415.
(5) Klauda, J. B.; Venable, R. M.; Freites, J. A.; O'Connor, J. W.; Tobias, D. J.; Mondragon-Ramirez, C.; Vorobyov, I.; MacKerell, A. D.; Pastor, R. W. Update of the CHARMM All-Atom Additive Force Field for Lipids: Validation on Six Lipid Types. *J. Phys. Chem. B* **2010**, *114*, 7830-7843.
(6) Jorgensen, W. L.; Chandrasekhar, J.; Madura, J. D.; Impey, R. W.; Klein, M. L. Comparison of Simple Potential Functions for Simulating Liquid Water. *J. Chem. Phys.* **1983**, *79*, 926-935.
(7) Darden, T.; York, D.; Pedersen, L. Particle Mesh Ewald: An N·log(N) Method for Ewald Sums in Large Systems. *J. Chem. Phys.* **1993**, *98*, 10089-10092.
(8) Hess, B.; Bekker, H.; Berendsen, H. J. C.; Fraaije, J. G. E. M. LINCS: A Linear Constraint Solver for Molecular Simulations. *J. Comput. Chem.* **1997**, *18*, 1463-1472.
(9) Nosé, S. A unified Formulation of the Constant Temperature Molecular Dynamics Methods. *J. Chem. Phys.* **1984**, *81*, 511-519.
(10) Parrinello, M.; Rahman, A. Polymorphic Transitions in Single Crystals: A New Molecular Dynamics Method. *J. Appl. Phys.* **1981**, *52*, 7182-7190.
(11) Souza, P. C. T.; Alessandri, R.; Barnoud, J.; Thallmair, S.; Faustino, I.; Grünewald, F.; Patmanidis, I.; Abdizadeh, H.; Bruininks, B. M. H.; Wassenaar, T. A.; et al. Martini 3: a General Purpose Force Field for Coarse-Grained Molecular Dynamics. *Nat. Methods* **2021**, *18*, 382-388.
(12) Kroon, P. C.; Grünewald, F.; Barnoud, J.; van Tilburg, M.; Souza, P. C. T.; Wassenaar, T. A.; Marrink, S.-J. Martinize2 and Vermouth: Unified Framework for Topology Generation. *arXiv e-prints* **2022**, arXiv:2212.01191.
(13) Wassenaar, T. A.; Ingólfsson, H. I.; Böckmann, R. A.; Tieleman, D. P.; Marrink, S. J. Computational Lipidomics with insane: A Versatile Tool for Generating Custom Membranes for Molecular Simulations. *J. Chem. Theory Comput.* **2015**, *11*, 2144-2155.
(14) Poma, A. B.; Cieplak, M.; Theodorakis, P. E. Combining the MARTINI and Structure-Based Coarse-Grained Approaches for the Molecular Dynamics Studies of Conformational Transitions in Proteins. *J. Chem. Theory Comput.* **2017**, *13*, 1366-1374.
(15) Periole, X.; Cavalli, M.; Marrink, S.-J.; Ceruso, M. A. Combining an Elastic Network With a Coarse-Grained Molecular Force Field: Structure, Dynamics, and Intermolecular Recognition. *J. Chem. Theory Comput.* **2009**, *5*, 2531-2543.
(16) Bussi, G.; Donadio, D.; Parrinello, M. Canonical Sampling Through velocity Rescaling. *J. Chem. Phys.* **2007**, *126*, 014101.

(17) de Jong, D. H.; Baoukina, S.; Ingólfsson, H. I.; Marrink, S. J. Martini Straight: Boosting Performance Using a Shorter Cutoff and GPUs. *Comput. Phys. Commun.* **2016**, *199*, 1-7.
(18) Grünewald, F.; Souza, P. C. T.; Abdizadeh, H.; Barnoud, J.; de Vries, A. H.; Marrink, S. J. Titratable Martini model for Constant pH Simulations. *J. Chem. Phys.* **2020**, *153*, 024118.
(19) Thurlkill, R. L.; Grimsley, G. R.; Scholtz, J. M.; Pace, C. N. pK Values of the Ionizable Groups of Proteins. *Protein Science* **2006**, *15*, 1214-1218.
(20) Isom, D. G.; Castañeda, C. A.; Cannon, B. R.; Velu, P. D.; García-Moreno E, B. Charges in the Hydrophobic Interior of Proteins. *Proc. Natl. Acad. Sci.* **2010**, *107*, 16096-16100.
(21) Van Gunsteren, W. F.; Berendsen, H. J. C. A Leap-frog Algorithm for Stochastic Dynamics. *Molecular Simulation* **1988**, *1*, 173-185.
(22) Wassenaar, T. A.; Pluhackova, K.; Böckmann, R. A.; Marrink, S. J.; Tieleman, D. P. Going Backward: A Flexible Geometric Approach to Reverse Transformation from Coarse Grained to Atomistic Models. *J. Chem. Theory Comput.* **2014**, *10*, 676-690.
(23) Barducci, A.; Bussi, G.; Parrinello, M. Well-Tempered Metadynamics: A Smoothly Converging and Tunable Free-Energy Method. *Phys. Rev. Lett.* **2008**, *100*, 020603.
(24) Johnston, J. M.; Wang, H.; Provasi, D.; Filizola, M. Assessing the Relative Stability of Dimer Interfaces in G Protein-Coupled Receptors. *PLoS Comput. Biol.* **2012**, *8*, e1002649.
(25) Tiwary, P.; Parrinello, M. A Time-Independent Free Energy Estimator for Metadynamics. *J. Phys. Chem. B* **2015**, *119*, 736-742.
(26) Tribello, G. A.; Bonomi, M.; Branduardi, D.; Camilloni, C.; Bussi, G. PLUMED 2: New Feathers for an Old Bird. *Comput. Phys. Commun.* **2014**, *185*, 604-613.
(27) Bonomi, M.; Bussi, G.; Camilloni, C.; Tribello, G. A.; Banáš, P.; Barducci, A.; Bernetti, M.; Bolhuis, P. G.; Bottaro, S.; Branduardi, D.; et al. Promoting Transparency and Reproducibility in Enhanced Molecular Simulations. *Nat. Methods* **2019**, *16*, 670-673.
(28) Daura, X.; Gademann, K.; Jaun, B.; Seebach, D.; van Gunsteren, W. F.; Mark, A. E. Peptide Folding: When Simulation Meets Experiment. *Angew. Chem., Int. Ed.* **1999**, *38*, 236-240.
(29) Liguori, N.; Campos, S. R. R.; Baptista, A. M.; Croce, R. Molecular Anatomy of Plant Photoprotective Switches: The Sensitivity of PsbS to the Environment, Residue by Residue. *J. Phys. Chem. Lett.* **2019**, *10*, 1737-1742.
